# The risk of cirrhosis in non-alcohol drinkers is greater in female than male betel nut chewers

**DOI:** 10.18632/oncotarget.23885

**Published:** 2018-01-03

**Authors:** Yu-Hua Chu, Lee Wang, Pei-Chieh Ko, Shou-Jen Lan, Yung-Po Liaw

**Affiliations:** ^1^ Department of Healthcare Administration, Asia University, Wufeng District, Taichung City, Taiwan; ^2^ Department of Public Health and Institute of Public Health, Chung Shan Medical University, Taichung, Taiwan; ^3^ Department of Medical Research, China Medical Hospital, China Medical University, Taichung, Taiwan; ^4^ Department of Family and Community Medicine, Chung Shan Medical University Hospital, Taichung, Taiwan

**Keywords:** cirrhosis, betel nut, non-alcohol drinker, public health, Taiwan

## Abstract

**Background and Aim:**

The association of betel nut with liver cirrhosis among alcohol drinkers has been clearly shown. However, very few studies have shown such an association among non-alcohol drinkers. The aim of this study was to assess the relationship between betel nut chewing and cirrhosis among non-alcohol drinkers.

**Materials and Methods:**

This study retrospectively analyzed data retrieved from the 2012 Adult Preventive Medical Services and the National Health Insurance Research Datasets in Taiwan. Participants’ information included physical examination and lifestyle, alongside laboratory tests. Betel nut chewers were grouped into three categories: never, occasional and frequent. Diseases were diagnosed using the International Classification of Diseases, Ninth Revision, Clinical Modification (ICD-9-CM). Initially, 1573024 adults aged 40 years and above who engaged in the free adult preventive medical services in 2012 were recruited. However, only 1065246 of them were included in the analysis. Chi-square test and logistic regression were used for the analyses.

**Results:**

After multivariable adjustments, there were significant relationships between cirrhosis and betel nut chewing in both sexes (P-trend < 0.0001). The risk of cirrhosis was greater in females than males. The odds ratios of cirrhosis in occasional and frequent female chewers were respectively 2.91; 95% C.I: 1.75–4.83 and 3.06; 95% C.I: 1.69–5. However, they were respectively 1.76; 95% C.I: 1.47–2.10 and 2.32; 95% C.I: 1.90–2.85 in occasional and frequent male chewers.

**Conclusions:**

This study demonstrated significant relationships between betel nut chewing and cirrhosis in both male and female non-alcohol drinkers. The risk of cirrhosis was greater in female than male chewers.

## INTRODUCTION

Liver cirrhosis is the 6th disease-related principal cause of death in Taiwan [1]. Advanced fibrotic changes in a chronically damaged liver could lead to cirrhosis [2, 3]. Betel nut chewing, alcoholism, smoking, and infections with hepatitis B virus (HBV) and hepatitis C virus (HCV) are common risk factors of liver cirrhosis [1, 4–8]. Women are generally less prone to liver cirrhosis/diseases than men [9–12]. However, some studies have revealed that they are more prone to alcohol-induced liver diseases than men [9, 13–15]. Betel nut chewing is a very common practice in Asia and is reported to be the fourth most popular psychoactive substance in the world [16]. A case-control study in the South of Taiwan found more than 10% of the population under study as lifetime betel nut chewers [17]. The betel nuts chewed in Taiwan are wrapped in piper betel leaves [4]. However, they are not mixed with tobacco [18, 19]. The association of betel nut chewing with liver cirrhosis among alcohol drinkers has been clearly shown [4–7]. The carcinogenicity and hepatotoxicity of betel nut have been linked to high concentrations of aflatoxin B1 (in the nuts) and safrole (in the Piper betel plant). Moreover, the carcinogenicity is also due to certain nitrosamines formed from arecal alkaloids in the mouth and stomach especially in an acidic medium as well as in the presence of bacterial nitrite products [4–7, 20]. Very few studies have focused on the association of betel nut with cirrhosis among non-alcohol drinkers. Moreover, the outcomes from those studies are still controversial. The aim of our study was therefore to assess the association between betel nut chewing and cirrhosis among male and female non-alcohol drinkers.

## MATERIALS AND METHODS

**Figure 1:**
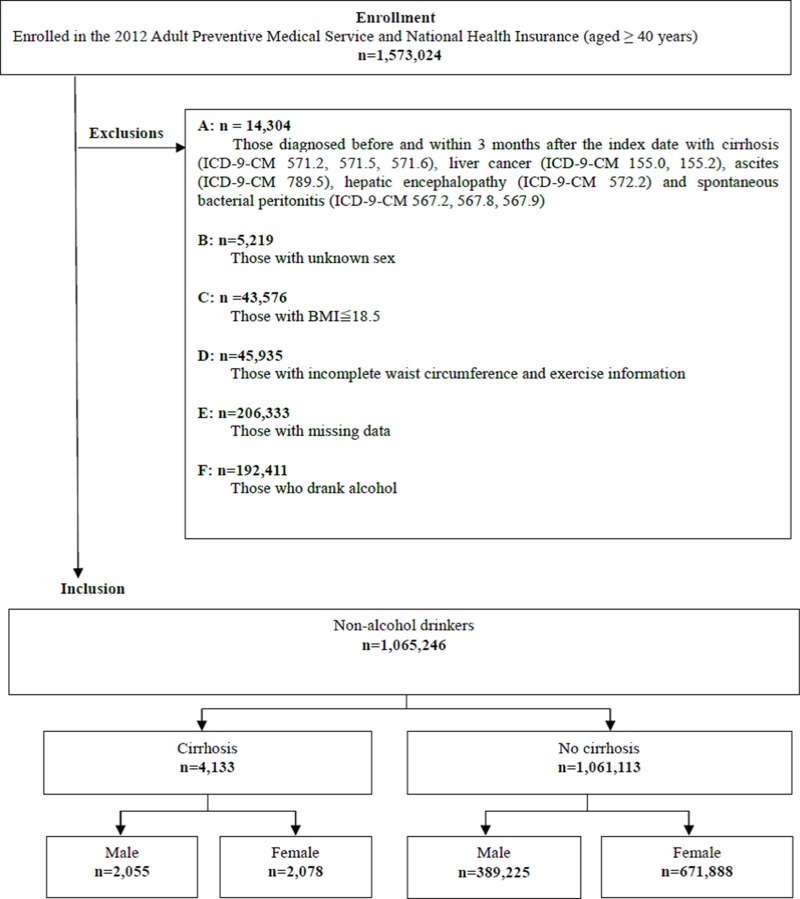
Study flow chart

This study retrospectively analyzed data retrieved from the 2012 Adult Preventive Medical Services and the National Health Insurance Research Datasets in Taiwan. These datasets are provided by the Health Promotion Administration (HPA) and the statistics department of the Ministry of Health and Welfare, respectively. Self-reported betel nut and alcohol consumption, as well as age and sex of participants were obtained from questionnaires contained in the 2012 and 2013 adult preventive medical service database. Based on information from the questionnaires, participants were defined as never, occasional and frequent betel nut chewers. Moreover, those who answered “no” to alcohol consumption were defined as non-alcohol drinkers while those who answered “yes” were alcohol-drinkers. Other information available in this database included physical examination (personal/family medical history, height, weight, blood pressure, among others) and lifestyle (nutrition, smoking, sexual behavior and exercise) alongside laboratory tests (complete blood count, fasting plasma sugar; liver, renal and lipid profiles as well as urinalysis). The adult preventive care service was initiated in 1996 by the Bureau of National Health Insurance (NHI) and has been providing free medical services for adults aged 40–65 and ≥ 65 years triennially and annually, respectively. The adult preventive medical services dataset was linked to the NHIRD using personal identification numbers of the participants which were protected for privacy reasons. Diseases were diagnosed using the International Classification of Diseases, Ninth Revision, Clinical Modification (ICD-9-CM). The date of examination for participation was the index date. Initially, 1573024 adults aged 40 years and above who engaged in the free adult preventive medical services in 2012 were recruited. However, 14304 of them who were diagnosed with cirrhosis (ICD-9-CM 571.2, 571.5, 571.6), liver cancer (ICD-9-CM 155.0, 155.2), ascites (ICD-9-CM 789.5), hepatic encephalopathy (ICD-9-CM 572.2) and spontaneous bacterial peritonitis (ICD-9-CM 567.2, 567.8, 567.9) before and within 3 months after the index date were excluded. In addition, those with unknown sex (*n* = 5219), BMI ≤ 18.5 (*n* = 43576), incomplete waist circumference and exercise information (*n* = 45935), missing data (*n* = 206333), as well as alcohol drinkers (*n* = 192411) were excluded. The final study sample included 1065246 non-alcohol drinkers (*n* = 4133) and without cirrhosis (*n* = 1061113). Those with cirrhosis comprised 2055 men and 2078 women (Figure 1). Basic characteristics of the participants in the various betel nut chewing groups were compared using the Chi-square test. The relationship between betel nut and cirrhosis was determined using logistic regression and the odds ratios (ORs) and confidence intervals (CIs) were reported. OR = 1.00 was interpreted as “no association between betel nut chewing and cirrhosis”, while OR < 1.00 and OR > 1.00 were interpreted as lower and higher risks, respectively. Odd ratios were considered significant if the confidence intervals did not cross 1.00. Confounding variables including smoking, age, body mass index (BMI), exercise, glutamate pyruvate transaminase (GPT), creatinine, estimated glomerular filtration rate (eGFR.), Glutamic Oxalic Transaminase (GOT), urinary proteins, lipoproteins including cholesterol (Cho), high density lipoproteins (HDL), low-density lipoproteins (LDL), Cho/HDL, LDL/HDL and comorbidities including metabolic syndrome, asthma, chronic obstructive pulmonary disease (COPD), tuberculosis (TB), diabetes mellitus (DM), acute coronary syndrome (ACS), cerebrovascular accidents (CVA), hypertension (HTN), hepatitis B virus (HBV) and hepatitis C virus (HCV) were adjusted for. Data analyses were performed using the SAS 9.3 statistical software (SAS Institute Inc., Cary, NC, USA). This study was approved by the institutional review board of Chung Shan Medical University Hospital.

## RESULTS

Table 1 shows the baseline characteristics of male and female non-alcohol drinkers by cirrhosis status. In terms of betel nut chewing, age, BMI, exercise, cholesterol, triglyceride, Cho/HDL, LDL/HDL, asthma, COPD and TB, the percentages of cirrhotic individuals were significantly different from those of non-cirrhotic individuals. These differences were observed in both males and females. However, significant differences were only observed in males (for smoking) and females (metabolic syndrome). Table 2 shows the odds ratios of cirrhosis among non-alcohol drinking betel nut chewers classified by gender. There were significant relationships between cirrhosis and betel nut in both males and females (P-trend < 0.0001) after multivariable adjustments. The risk of cirrhosis was greater in the frequent than occasional chewers. Notwithstanding, the odds of cirrhosis were greater among female chewers than their male counterparts. Among the females, the odds of cirrhosis in occasional and frequent chewers were respectively 2.91 (95% C.I: 1.75–4.83) and 3.06 (95% C.I: 1.69–5.57). Among the males, the odds in occasional and frequent chewers were respectively 1.76 (95% C.I: 1.47–2.10) and 2.32 (95% C.I:1.90–2.85). The odds of cirrhosis in males and females with HBV were 2.51 (95% CI: 1.94–3.24) and 3.16 (95% CI: 1.41–7.10), respectively while those in males and females with HCV were 2.85 (95% CI: 2.09–3.87) and 2.70 (95% CI: 1.18–6.20), respectively. Furthermore, there were significant associations between cirrhosis and diabetes mellitus in both males and females. However, the association between cirrhosis and hypertension was only significant in males.

**Table 1:** Baseline characteristics of male and female non-alcohol drinkers by cirrhosis status

	Male			Female		
	Cirrhosis	No cirrhosis	*P*	Cirrhosis	No cirrhosis	*P*
	*n* (%)	*n* (%)		*n* (%)	*n* (%)	
**Betel nut**			0.0003			< .0001
Never	1980 (96.35)	379844 (97.59)		2048 (98.56)	668869 (99.55)	
Occasional	46 (2.24)	6467 (1.66)		18 (0.87)	2001 (0.30)	
Frequent	29 (1.41)	2914 (0.75)		12 (0.58)	1018 (0.15)	
**Smoking (pack/day)**			0.0361			0.1623
Never	1734 (84.38)	335535 (86.21)		2051 (98.70)	660433 (98.30)	
≤ 1	252 (12.26)	43220 (11.10)		21 (1.01)	9981 (1.49)	
> 1	69 (3.36)	10470 (2.69)		6 (0.29)	1474 (0.22)	
**Age (years)**			0.0003			< .0001
40 ≤ Age<60	1821 (88.61)	332579 (85.45)		1832 (88.16)	560349 (83.40)	
60 ≤ Age<80	153 (7.45)	36643 (9.41)		177 (8.52)	75072 (11.17)	
80 ≤ Age	81 (3.94)	20003 (5.14)		69 (3.32)	36467 (5.43)	
**BMI (kg/m^2^)**			0.0019			< .0001
Normal	874 (42.53)	153450 (39.42)		747 (35.95)	309021 (45.99)	
Overweight	650 (31.63)	136928 (35.18)		632 (30.41)	195168 (29.05)	
Obesity	531 (25.84)	98847 (25.40)		699 (33.64)	167699 (24.96)	
**Exercise (hours/week)**			0.0006			< .0001
No	1110 (54.01)	196485 (50.48)		1217 (58.57)	353454 (52.61)	
< 2.5	693 (33.72)	134809 (34.64)		685 (32.96)	233536 (34.76)	
≥ 2.5	252 (12.26)	57931 (14.88)		176 (8.47)	84898 (12.64)	
**Cholesterol (mg/dl)**			<.0001			< .0001
Cho < 200	1603 (78.00)	239780 (61.60)		1552 (74.69)	328006 (48.82)	
Cho ≥ 200	452 (22.00)	149445 (38.40)		526 (25.31)	343882 (51.18)	
**Triglyceride (mg/dl)**			<.0001			< .0001
TG < 150	1620 (78.83)	267558 (68.74)		1680 (80.85)	487090 (72.50)	
TG ≥ 150	435 (21.17)	121667 (31.26)		398 (19.15)	184798 (27.50)	
**Cho/HDL**			<.0001			0.0002
Cho/HDL < 5	1739 (84.62)	301594 (77.49)		1883 (90.62)	591105 (87.98)	
Cho/HDL ≥ 5	316 (15.38)	87631 (22.51)		195 (9.38)	80783 (12.02)	
**LDL/HDL**			<.0001			< .0001
LDL/HDL < 3	1631 (79.37)	275793 (70.86)		1808 (87.01)	555540 (82.68)	
LDL/HDL ≥ 3	424 (20.63)	113432 (29.14)		270 (12.99)	116348 (17.32)	
**Comorbidity**						
Metabolic syndrome	837 (40.73)	150860 (38.76)	0.0674	1150 (55.34)	285378 (42.47)	< .0001
Asthma	99 (4.82)	15182 (3.90)	0.0324	133 (6.40)	25026 (3.72)	< .0001
COPD	260 (12.65)	33031 (8.49)	<.0001	188 (9.05)	30535 (4.54)	< .0001
TB	26 (1.27)	2814 (0.72)	0.0039	10 (0.48)	1436 (0.21)	0.0085
DM	795 (38.69)	96388 (24.76)	<.0001	813 (39.12)	141666 (21.08)	< .0001
ACS	296 (14.40)	47797 (12.28)	0.0034	294 (14.15)	60182 (8.96)	< .0001
CVA	213 (10.36)	30964 (7.96)	<.0001	162 (7.8)	32146 (4.78)	< .0001
HTN	1339 (65.16)	236111 (60.66)	<.0001	1465 (70.5)	365019 (54.33)	< .0001
HBV	248 (12.07)	10160 (2.61)	<.0001	145 (6.98)	12226 (1.82)	< .0001
HCV	235 (11.44)	5352 (1.38)	<.0001	368 (17.71)	9039 (1.35)	< .0001

**Table 2:** Odds ratios of cirrhosis among non-alcohol drinkers by gender

	Male			Female		
	OR	95% C.I.	*P*	OR	95% C.I.	*P*
**Betel nut** **(Reference = Never)**	1.00			1.00		
Occasional	1.76	1.47–2.10	<.0001	2.91	1.75–4.83	<.0001
Frequent	2.32	1.90–2.85	<.0001	3.06	1.69–5.57	0.0002
**Age (years)** **(Reference = 40** ≤ **Age<60)**	1.00			1.00		
60 ≤ Age<80	1.49	1.28–1.74	<.0001	1.23	0.78–1.94	0.3836
80 ≤ Age	1.50	1.06–2.11	0.0205	2.79	1.14–6.83	0.0246
**Exercise (hours/week)** **(Reference = No)**	1.00			1.00		
< 2.5	0.77	0.66–0.89	0.0005	0.73	0.48–1.11	0.1389
≥ 2.5	0.79	0.64–0.98	0.0327	0.26	0.08–0.83	0.0228
**Cho/HDL** **(Reference < 5)**	1.00			1.00		
Cho/HDL ≥ 5	0.82	0.65–1.04	0.0966	1.21	0.53–2.75	0.6453
**LDL/HDL** **(Reference < 3)**	1.00			1.00		
LDL/HDL ≥ 3	0.74	0.59–0.92	0.0079	0.30	0.12–0.74	0.0093
**Comorbidity**						
Metabolic syndrome	1.30	1.11–1.54	0.0017	1.20	0.74–1.96	0.4603
Asthma	0.91	0.61–1.36	0.6456	0.90	0.38–2.14	0.8137
COPD	1.17	0.88–1.55	0.2748	0.89	0.38–2.06	0.7850
TB	1.43	0.71–2.88	0.3176	6.44	1.80–23.00	0.0041
DM	1.65	1.42–1.92	<.0001	1.82	1.16–2.87	0.0093
ACS	0.90	0.69–1.18	0.4489	1.37	0.67–2.83	0.3894
CVA	0.87	0.61–1.25	0.4584	0.29	0.04–2.10	0.2196
HTN	1.23	1.06–1.43	0.0072	1.05	0.67–1.65	0.8395
HBV	2.51	1.94–3.24	<.0001	3.16	1.41–7.10	0.0053
HCV	2.85	2.09–3.87	<.0001	2.70	1.18–6.20	0.0189

Multivariate analysis included betel nut chewing, smoking, age, body mass index (BMI), exercise, glutamate pyruvate transaminase (GPT), creatinine, estimated glomerular filtration rate (eGFR.), Glutamic Oxalic Transaminase (GOT), urinary proteins, lipoproteins including cholesterol (Cho), high density lipoproteins (HDL), low-density lipoproteins (LDL), Cho/HDL, LDL/HDL and comorbidities including metabolic syndrome, asthma, chronic obstructive pulmonary disease (COPD), tuberculosis (TB), diabetes mellitus (DM), acute coronary syndrome (ACS), cerebrovascular accidents (CVA), hypertension (HTN), hepatitis B virus (HBV) and hepatitis C virus (HCV).

## DISCUSSION

To our knowledge, this study is the first to demonstrate a relationship between betel nut chewing and cirrhosis in both male and female non-alcohol drinkers in Taiwan. Early epidemiological studies have shown that betel nut chewing is an important risk factor for liver cirrhosis [1, 4–8]. However, these studies did not clearly focus on non-alcohol drinkers. The relationship between betel nut and cirrhosis is yet to be fully understood. Betel nut chewers have been reported to have a higher likelihood of engaging in more risky behaviors like the use of illicit drugs which can expose them to potential risk factors of cirrhosis like HBV [6, 21]. Moreover, betel nut is toxic to the liver and therefore increases alkaline phosphates and transaminases in the serum [22–24]. In animal studies, it has been shown to be damaging to DNA, causing cell death and necrosis leading to abnormal liver cell growth [23, 24]. The toxic and abnormal nature of the liver can interfere with its detoxification capacity exposing it to more carcinogenic substances. Some studies have shown that women have lower risks of liver cirrhosis and diseases than men [9–12]. One possible explanation is that estrogen, a typical female hormone inhibits the formation of carcinogenic substances while androgen, a typical male hormone stimulates liver cirrhosis or hepatocellular carcinoma [9–12, 25]. On the contrary, women had a higher risk when compared to men in the current study as well as some other studies [9, 13–15] even though other studies focused on alcohol-induced cirrhosis. Why the risk of cirrhosis was higher in women than men in this study remains unclear and deserves further investigations. This might be because the incidence rate of cirrhosis is lower in non-alcohol drinking females. Because of this, the relative index (OR) is bound to be higher while that in males whose incidence rate is higher is lower. The differences between our study and previous ones could possibly be as a result of different sample sizes, study designs, as well as the variables involved especially alcohol consumption. In our study, there were significant associations of cirrhosis with DM, HBV, and HCV in both males and females. These are consistent with previous studies [26–29]. However, the association of cirrhosis with hypertension was only significant among the males. Studies describing the association between hypertension and cirrhosis are limited [30]. However, the association of portal hypertension (high blood pressure in the hepatic portal system) and cirrhosis has been largely reported [31, 32].

Our study was not without any limitations. First, its cross-sectional nature prevented us from making causal inferences. Second, we did not have the information about the different stages of cirrhosis that could allow us to determine the relationship at these stages. Third, only a small proportion of the study participants were betel nut chewers because we included only non-alcohol drinkers. Even though the sample of betel chewers is small, it is still very valuable and we used the 95% confidence interval to show the precision of the estimates.

## CONCLUSIONS

In conclusion, our study demonstrates that betel nut chewing is a risk factor of cirrhosis in non-alcohol drinkers and women are more at risk than men. This study serves as a good tool in encouraging people not only to quit smoking and alcohol drinking but to also quit betel nut chewing in order to reduce their chances of developing cirrhosis. There is a need for further studies to re-investigate this association.

## Abbreviations

MOST: Ministry of Science and Technology
ICD-9-CM: International Classification of Diseases, Ninth Revision, Clinical Modification
C.I: confidence interval
OR: odds ratio
HBV: hepatitis B
HCV: hepatitis C
HPA: Health Promotion Administration
NHI: National Health Insurance
BMI: body mass index
GPT: glutamate pyruvate transaminase
eGFR: estimated glomerular filtration rate
GOT: glutamic oxalic transaminase
Cho: cholesterol
HDL: high density lipoproteins
LDL: low density lipoproteins
COPD: chronic obstructive pulmonary disease
TB: tuberculosis
DM: diabetes mellitus
ACS: acute coronary syndrome
CVA: cerebrovascular accidents
HTN: hypertension
